# Effect of *E. cava* and *C. indicum* Complex Extract on Phorbol 12-Myristate 13-Acetate (PMA)-Stimulated Inflammatory Response in Human Pulmonary Epithelial Cells and Particulate Matter (PM)_2.5_-Induced Pulmonary Inflammation in Mice

**DOI:** 10.3390/pharmaceutics15112621

**Published:** 2023-11-13

**Authors:** Sung-Gyu Lee, Chan-Hwi Park, Hyun Kang

**Affiliations:** Department of Medical Laboratory Science, College of Health Science, Dankook University, Cheonan-si 31116, Chungnam, Republic of Korea; sung-gyu@dankook.ac.kr (S.-G.L.); cksgnl1014@naver.com (C.-H.P.)

**Keywords:** inflammation, fine particulate matter, ED, *MUC5AC*, A549

## Abstract

This study explores the potential of a natural composite formulation known as ED, consisting of *Ecklonia cava* (*E. cava,* family: Lessoniaceae) and *Chrysanthemum indicum* Linne (*C. indicum*, family: Asteraceae), in alleviating lung inflammation induced by fine particulate matter (PM_2.5_). Initial assessments confirmed that neither ED nor one of its components, dieckol, exhibited cytotoxic effects on A549 cells. Subsequently, the impact of ED and dieckol on *MUC5AC* gene expression in A549 cells stimulated by phorbol 12-myristate 13-acetate (PMA) was investigated, revealing promising results that demonstrated a dose-dependent inhibition of *MUC5AC* gene expression. The study also delves into the underlying mechanisms, demonstrating that ED and dieckol effectively suppressed the phosphorylation of mitogen-activated protein kinases (MAPKs), including JNK, ERK, and p38, which are known to be involved in the regulation of *MUC5AC* gene expression. In in vivo experiments using a PM_2.5_-induced pulmonary inflammation mouse model, the research findings showed that ED mitigated cellular accumulation in the airways, leading to a significant reduction in the total cell count in bronchoalveolar lavage fluid (BALF). Moreover, ED exhibited protective effects against PM_2.5_-induced pulmonary damage, characterized by reduced inflammatory cell infiltration and decreased mucus secretion in pulmonary tissues. Additionally, ED’s anti-inflammatory properties were evident in its ability to decrease the levels of key inflammatory cytokines, *TNF-α* and *IL-6*, both in the serum and lung tissue of the PM_2.5_-induced pulmonary inflammation mouse model. These findings suggest the potential of ED as a therapeutic agent for inflammatory respiratory diseases.

## 1. Introduction

Due to a rapid increase in fine particulate matter (PM), an air pollutant, respiratory diseases are on the rise not only in South Korea, China, and Japan but also in various other East Asian countries [1]. According to a 2016 report by the Organization for Economic Cooperation and Development (OECD), South Korea has the highest premature mortality rate among OECD member countries due to air pollution caused by fine particulate matter and ozone [2]. PM refers to particles smaller than 10 μm in diameter that cannot be visually identified [3]. PM consists of various airborne particles, including chemical toxic substances from automobile emissions, industrial activities, such as sulfur oxides, lead (Pb), and carbon monoxide (CO). Particularly, diesel exhaust particles (DEPs) are known to exacerbate asthma [4].

Inhalation of PM reaches the human airways and pulmonary cells, inducing inflammation, causing severe pulmonary damage, secondary cardiac impairments, and leading to various respiratory diseases [5]. Diseases caused by PM are known to affect not only respiratory conditions but also a diverse range of ailments including circulatory disorders, dementia, and strokes [6,7]. Over 70% of inhaled PM accumulates in the lower respiratory tract, with approximately 22% reaching the pulmonary cells. The accumulated PM affects the epithelial cells in the airways and pulmonary tissues, causing local tissue damage and triggering an inflammatory response due to oxidative stress [8]. Chronic inflammation can progress to allergic alveolitis, chronic bronchitis, and other conditions [9]. Chronic Obstructive Pulmonary Disease (COPD), a representative chronic respiratory disease caused by PM, is a condition where the pulmonary function is impaired due to abnormal inflammatory responses in the pulmonary system [10]. The pharmaceutical treatment for COPD primarily involves bronchodilators that improve smooth muscle tension in the airways, including anticholinergics, theophylline, and beta-2 agonists. However, direct evidence of these medications improving mortality rates is still lacking [11]. From a long-term perspective, there is a need for the development of safe and effective treatments with relatively few side effects.

Mucus secretion is essential for appropriate mucociliary function and maintaining homeostasis in the airways [12]. Mucus in the airways entraps inhaled dust particles, chemical substances, and microbes. However, excessive mucus production and accumulation within the airway lumen are pathological symptoms associated with various chronic respiratory conditions [13]. Airway obstruction due to mucus is a major contributor to morbidity and mortality in patients with chronic respiratory conditions [14]. To date, 20 different Mucin (MUC) genes have been identified [15]. In normal human airways, *MUC5AC* is primarily expressed on surface goblet epithelial cells [16]. Previous reports have demonstrated abnormal elevation and accumulation of *MUC5AC* in the airway secretions of patients with pulmonary diseases such as asthma, COPD, and cystic fibrosis [17].

Concerns about respiratory disorders caused by PM are increasing. Accordingly, there is ongoing drug development to prevent or treat respiratory damage caused by PM [1]. Bioactive compounds derived from natural sources exhibit excellent anti-inflammatory and antioxidant activities with relatively low side effects [18]. Therefore, there is growing interest in the development of respiratory protectants using natural compounds in a PM_2.5_-induced pulmonary inflammation model [19,20].

ED is an acknowledged extract developed from a combination of *Ecklonia cava* (*E. cava*) and *Chrysanthemum indicum* Linne (*C. indicum*) for the treatment of respiratory diseases. Notably, one of the constituents found within ED, dieckol, is a phenolic compound isolated from marine brown algae. Given the rich biodiversity and abundant sources of bioactive compounds in marine environments, considerable efforts have been made to explore functional components from marine algae [21,22]. Dieckol, a polyphenol extracted from certain brown algae, including *E. cava*, has been reported to possess various pharmacological properties such as antiviral, antioxidant, anti-inflammatory, and anticancer effects [23,24,25,26]. Recent studies have reported the protective effects of dieckol against oxidative stress induced by PM and in vitro inflammatory responses in dendritic cells and skin keratinocytes [27,28]. However, there is a scarcity of reports regarding the impact of dieckol on respiratory-related conditions.

We previously reported that ED demonstrated a potent effect in improving asthma by inhibiting inflammatory cytokine levels in pulmonary tissues and suppressing the number of inflammatory cells in the bronchoalveolar lavage fluid (BALF) in an ovalbumin (OVA)-induced asthmatic mouse model. Additionally, ED suppressed serum interleukin (IL)-6 levels [29]. Therefore, we investigated whether ED has a protective effect against excessive mucus secretion and pulmonary inflammation induced by PM_2.5_ using a *MUC5AC* gene overexpression cell model and a PM_2.5_-induced pulmonary inflammation model. In animal models, a comparison was conducted with Bronpass tablets, a medication for acute bronchitis, which is manufactured using seven different herbal ingredients. We obtained histological data through pulmonary tissue staining and analyzed molecular pathological markers of cytokines involved in inflammation. Here, we demonstrate that ED has significant potential as an anti-inflammatory agent for use in conventional therapies for pulmonary inflammation induced by PM_2.5_.

## 2. Materials and Methods

### 2.1. Materials

#### 2.1.1. Cells and Chemicals

The A549 pulmonary epithelial cell line was obtained from the Korean Cell Line Bank (KCLB; Seoul, Republic of Korea). The cells were cultured in RPMI 1640 medium (Cat No. 11875-093, Gibco-BRL, Carlsbad, CA, USA) containing 10% fetal bovine serum (FBS; Cat No. 16000044, Gibco-BRL), 100 U/mL penicillin (Cat No. 15140122, Gibco-BRL), and 100 mg/mL streptomycin (Gibco-BRL). The BCA Protein Assay Kit (Cat No. 23225) and TRIzol reagent (Cat No. 15596026) were purchased from Thermo Scientific (Rockford, IL, USA). All antibodies used in the experiments were purchased from Cell Signaling Technology (Danvers, MA, USA). IL-6 (Cat No. M6000B-1) and TNF-α (Cat No. MTA00B-1) enzyme-linked immunosorbent assay (ELISA) kits were purchased from R&D Systems (Minneapolis, MN, USA). Other reagents used in the experiments were purchased from Sigma-Aldrich (St. Louis, MO, USA).

#### 2.1.2. Preparation of ED

ED, a mixture of *C. indicum* ethanolic extract and *E. cava* concentrate, prepared using the protocol described previously, was used in the experiments. The ED used in the experiments was manufactured by S&D Co., Ltd. (Recognition No. 2015-6, Cheongju-si, Chungcheongbuk-do, Republic of Korea). *E. cava* ethanol extract was derived from the ethanol extract of the brown alga *E. cava* and subsequently processed to achieve a dieckol concentration of 60 mg/g. *C. indicum* was extracted, filtered, and concentrated. Following this, *E. cava* extract and an excipient (dextrin) were added and mixed to prepare ED (Lot. SD-ED-001) using a spray dryer (inlet temperature: 185–210 °C; outlet temperature: 85–98 °C) for use in the experiment. The ED used in the experiments contained the active compound dieckol (S&D Co., Ltd.) at a concentration of 9.5 mg/g [30].

#### 2.1.3. Preparation of PM_2.5_

Urban PM_2.5_ from the National Institute of Standards and Technology (NIST) (SRM 1648a) was procured from Sigma-Aldrich. The atmospheric pollutant (SRM 1648a), which is commonly used as a standard for PM research, has well-defined and stable components. To use in the experiments, PM_2.5_ was dissolved in phosphate-buffered saline (PBS) at a concentration of 50 mg/kg.

### 2.2. Methods

#### 2.2.1. A549 Cell Culture

A549 cells were cultured in RPMI 1640 medium containing 10% FBS, following the culture protocol described in a previous study [22]. A549 cells were seeded at a density of 1 × 10^5^ cells/well in a 6-well plate and cultured at 37 °C in a 5% CO_2_ atmosphere for 24 h. After removing the medium, the cells were pre-treated with various concentrations of ED (10, 20, 40, and 80 μg/mL) and dieckol (5, 10, 20, and 40 μg/mL) for 1 h, following which the medium was replaced with serum-free medium. Subsequently, the medium containing Phorbol 12-myristate 13-acetate (PMA, 10 nM) was added, and the cells were further cultured under the same conditions for 30 min or 24 h.

#### 2.2.2. Cell Viability Assay

Cell toxicity was measured using the 3-(4,5-Dimethylthiazol-2-yl)-2,5-diphenyltetrazolium bromide (MTT) reduction method. A549 cells were seeded at a density of 1 × 10^4^ cells/well in a 96-well plate and cultured at 37 °C in a 5% CO_2_ atmosphere for 24 h. The medium was then removed, and serum-free medium was added. The cells were treated with various concentrations of ED (10, 20, 40, and 80 μg/mL) and dieckol (5, 10, 20, and 40 μg/mL) and cultured under the same conditions for 24 h. Cell viability was determined using the MTT assay.

#### 2.2.3. Reverse Transcription-Polymerase Chain Reaction (RT-PCR)

Total RNA from cells and pulmonary tissues was extracted using TRIzol reagent (Thermo Fisher Scientific, Rockford, IL, USA). Subsequently, cDNA was synthesized using the RT premix kit (Bioneer, Daejeon, Republic of Korea). The synthesized cDNA was then subjected to PCR by reacting it with forward and reverse primers at a concentration of 20 pmol/mL in PCR premix (Bioneer). The sequences of the primers used are presented in Table 1, and the PCR products obtained were analyzed by electrophoresis on a 1.2% agarose gel to confirm the bands. 

#### 2.2.4. Western Blot Analysis

After culturing, the A549 cell pellets were mixed with RIPA lysis buffer and incubated with agitation for 30 min to extract proteins. The extracted proteins were quantified using the BCA Protein Assay Kit. A total of 20 μg of proteins from each sample were subjected to 10% SDS polyacrylamide gel electrophoresis and then transferred to PVDF membranes. The membranes were blocked with 5% skim milk for 30 min. Primary rabbit monoclonal antibodies (Cell Signaling Technology, Danvers, MA, USA) against p-JNK, p-ERK, p-p38, and β-Actin were then added at a 1:1000 dilution and incubated overnight at 4 °C. After washing the membrane with Tris-buffered saline supplemented with 0.1% Tween 20 (TBST), it was incubated with anti-rabbit HRP-linked antibody at room temperature (RT) for 2 h. The immune-reactive bands were visualized using a chemiluminescent imaging system (WSE-6100 Luminograph, ATTO, Tokyo, Japan) with ECL substrate (Bio-Rad, Hercules, CA, USA). Densitometric analysis was performed using Image J software (Version 1.53t, U.S. National Institutes of Health, Bethesda, MD, USA).

#### 2.2.5. PM_2.5_-Induced Pulmonary Inflammation Mouse Model and Experimental Design

**Figure 1:**
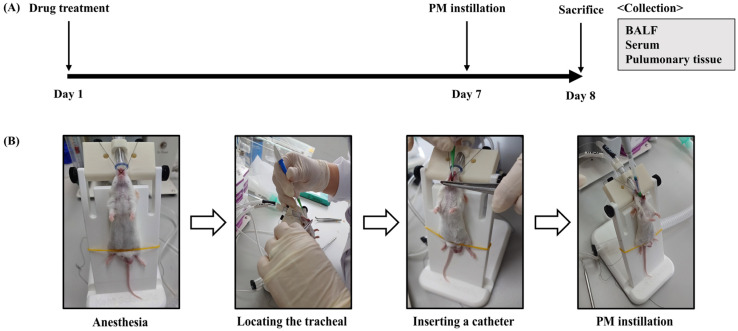
PM_2.5_-induced pulmonary inflammation mouse model preparation and experimental schedule. (**A**) ED and Bronpass tablets were orally administered once daily for 8 days starting on day 1. BALB/c mice were instilled with PM_2.5_ into the tracheal on the 7th day. **(B**) Using an endotracheal intubation kit, PM_2.5_ solution was slowly instilled through the tracheal.

Male BALB/c mice, approximately 6 weeks old and weighing about 20 ± 2 g, were obtained from DaeHan BioLink (DBL, Eumsung-gun, Chungcheongbuk-do, Republic of Korea). The mice were housed under consistent environmental conditions with a temperature of approximately 23 ± 2 °C, relative humidity of 50 ± 5%, and a regular 12 h light–dark cycle for acclimatization. The mice were provided with unlimited access to food and water. After a one-week acclimation period, the mice were randomly assigned to five groups: control group (Normal, *n* = 5), PM_2.5_ treatment group (PM_2.5_, *n* = 5), PM_2.5_ with 150 mg/kg ED treatment (ED150, *n* = 5), PM_2.5_ with 300 mg/kg ED treatment (ED300, *n* = 5), and PM_2.5_ with 300 mg/kg Bronpass Tablet (p.o., Hanlim Pharm. Co., Ltd., Yongin-si, Republic of Korea) treatment (Bronpass300, *n* = 5; used as a positive control). To induce pulmonary inflammation, PM_2.5_ dissolved in PBS (50 mg/kg, 50 μL) was administered intratracheally to the mice on day 7. ED and Bronpass Tablets were orally administered to the mice from day 0 to day 8, diluted in distilled water (DW). On day 8, the mice were euthanized using isoflurane, and BALF, blood, and pulmonary tissue samples were collected for further analysis (Figure 1). All animal experiments were conducted after obtaining approval from the Dankook University Institutional Animal Care and Use Committee (DKU IACUC Approval No: DKU-23-049).

#### 2.2.6. Analysis of Cell Counting in BALF

To analyze the cell count in the BALF, we collected the fluid according to the previous method [21]. The BALF samples were centrifuged to pellet the cells. The pelleted cells were resuspended in PBS, and the total cell count was measured via staining with methylene blue. Additionally, Wright–Giemsa staining was performed to examine the cellular morphology indicative of inflammatory cells.

#### 2.2.7. Histopathological Analysis

The pulmonary tissue was fixed in 10% formalin solution, embedded in paraffin, sectioned into 5 μm slices, and stained with hematoxylin and eosin (H&E) as well as periodic acid-Schiff (PAS) [31]. Subsequently, pathological changes in the pulmonary tissue were observed using an optical microscope (Olympus, Tokyo, Japan).

#### 2.2.8. ELISA Analysis for TNF-α and IL-6 in Serum

The levels of TNF-α and IL-6 in the serum were measured according to the manufacturer’s instructions using ELISA kits. All measurements were performed in duplicate, and the optical density of each well was read at 450 nm using a microplate reader (Bio-Rad).

#### 2.2.9. Statistical Analysis

The data are presented as mean ± standard deviation (SD). Statistical significance was analyzed using analysis of variance (ANOVA). *p* values < 0.05 were considered statistically significant.

## 3. Results

### 3.1. Effects of ED and Dieckol on MUC5AC Gene Expression in PMA-Stimulated A549 Cells

**Figure 2:**
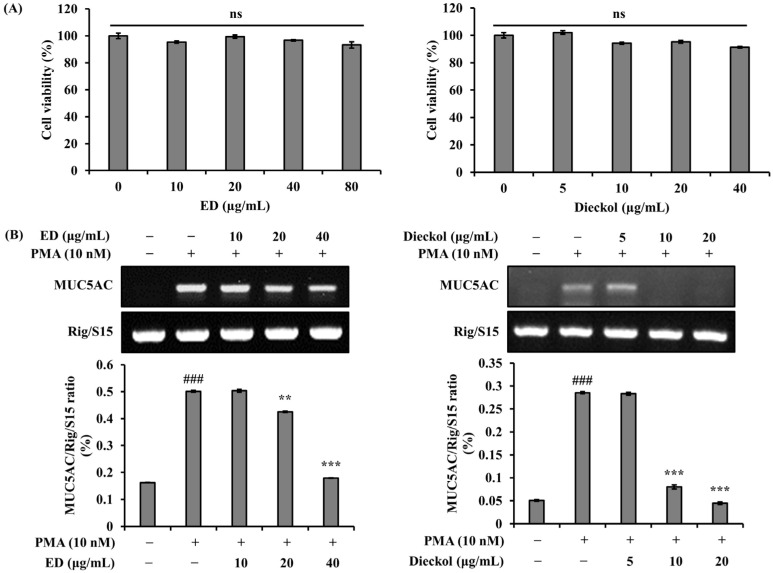
Effects of cell viability and *MUC5AC* mRNA levels of ED and dieckol. (**A**) In a 96-well plate, 1 × 10^4^ cells per well were seeded. After 24 h, ED and dieckol were treated for another 24 h with serum-free medium. Viability was measured using MTT assay. (**B**) RT-PCRanalysis using *Rig/S15* as the loading control was performed for measurement of *MUC5AC* mRNA expression in A549 cells. After ED and dieckol pre-treatment for 30 min, cells were PMA-stimulated for 24 h. The relative mRNA levels of *MUC5AC* were quantified using the Image J program. The average value of three independent experiments is shown. All data are expressed as the mean ± SD of the experiment. ### *p* < 0.001 compared to the control group. ** *p* < 0.01 and *** *p* < 0.001, compared to the PMA control group. ns: not statistically significant.

Prior to investigating the effects of ED and dieckol on *MUC5AC* gene expression, we measured the toxicity of ED and dieckol in A549 cells. The cells were treated with various concentrations of ED (10, 20, 40, and 80 μg/mL) and dieckol (5, 10, 20, and 40 μg/mL) and incubated for 24 h, followed by MTT analysis. According to the results, ED and dieckol showed no cytotoxicity at all tested concentrations (Figure 2A). We examined whether ED and dieckol had the ability to regulate *MUC5AC* gene expression in PMA-stimulated A549 cells. We pre-treated the cells with various concentrations of ED (10, 20, and 40 μg/mL) and dieckol (5, 10, and 20 μg/mL) for 30 min and measured the modulation of *MUC5AC* gene expression in cells stimulated with PMA for 24 h. The results showed that ED and dieckol dose-dependently inhibited *MUC5AC* gene expression (Figure 2B).

### 3.2. Effect of ED and Dieckol on the Phosphorylation of MAPKs in PMA-Stimulated A549 Cells

**Figure 3:**
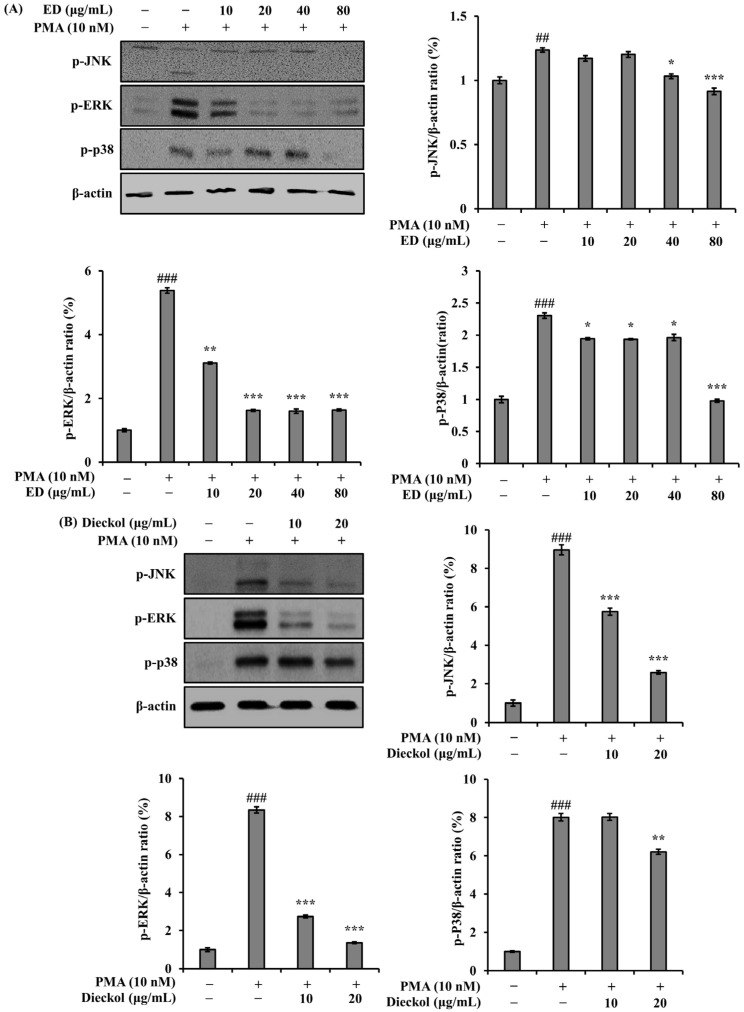
Effect of ED and dieckol on the phosphorylation of MAPKs in PMA-stimulated A549 cells. (**A**) ED and (**B**) dieckol pre-treatment for 30 min; cells were PMA-stimulated for 30 min. β-actin were detected and used as internal controls. The relative protein levels of p-JNK, p-ERK, and p-p38 were quantified using the Image J program and normalized to β-actin. The average value of three independent experiments is shown. All data are expressed as the mean ± SD of the experiment. ## *p* < 0.01 and ### *p* < 0.001 compared to the control group; * *p* < 0.05, ** *p* < 0.01, and *** *p* < 0.001 compared to the PMA control group. See also Figure S1.

To elucidate the mechanism by which *MUC5AC* gene expression is inhibited by ED and dieckol in PMA-stimulated A549 cells, we investigated the activation of specific mitogen activated protein kinases (MAPKs) in A549 cells after PMA stimulation. PMA activated phosphorylation of several signaling transducers, including JNK, ERK, and p38, known to be involved in *MUC5AC* gene expression. The phosphorylation of JNK, ERK, and p38 was significantly (*p* < 0.001) inhibited by ED and dieckol (Figure 3).

### 3.3. Effect of ED on Cells in BALF in the PM_2.5_-Induced Pulmonary Inflammation Mouse Model

**Figure 4:**
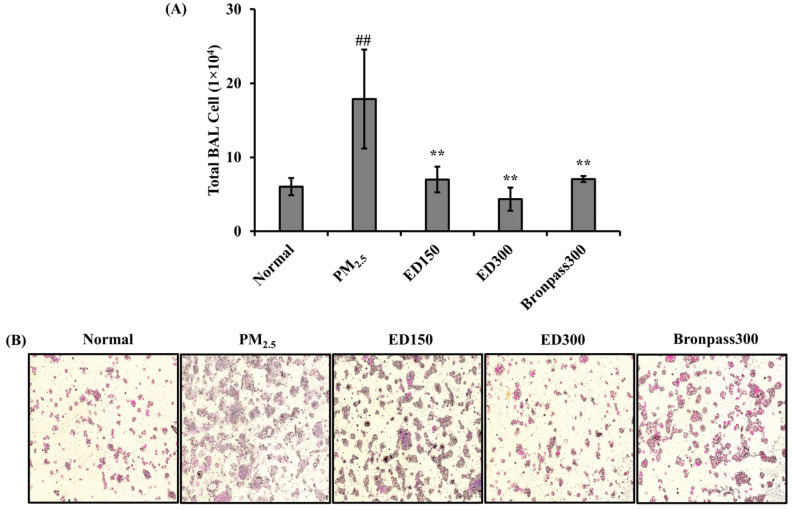
Effects of ED treatment on total cell count in PM_2.5_-induced pulmonary inflammation mouse model. (**A**) BALF was centrifuged, and the pellet was assessed for total cell count using the Trypan blue dye exclusion test. (**B**) Wright–Giemsa staining (magnification ×200) of cells in BALF. All results are shown as the mean ± SD (*n* = 5 per group). ## *p* < 0.01, compared to the normal group. ** *p* < 0.01, compared to the PM_2.5_ group.

Cellular accumulation in the airways is a characteristic feature of inflammatory respiratory diseases. Recent studies utilizing sputum induction or BAL techniques to measure and characterize pulmonary inflammation in patients with inflammatory respiratory diseases show a substantial number of cases exhibiting an increased total cell count and inflammatory cells in BALF [32]. The results of the total cell count and microscopic observation of cells in BALF of PM_2.5_-induced pulmonary inflammation mice are presented in Figure 4 to assess this aspect. The total cell count, as observed through Wright–Giemsa staining in BALF, significantly increased in the PM_2.5_ group (## *p* < 0.01). Conversely, a concentration-dependent decrease in the total cell count was observed in the ED150, ED300, and Bronpass300 groups compared to the PM_2.5_ group (** *p* < 0.01) (Figure 4A). The results from Wright–Giemsa staining showed a trend of decreased inflammatory cells in the ED groups compared to the PM_2.5_ group (Figure 4B).

### 3.4. Effect of ED on Pulmonary Damage in the PM_2.5_-Induced Pulmonary Inflammation Mouse Model

**Figure 5:**
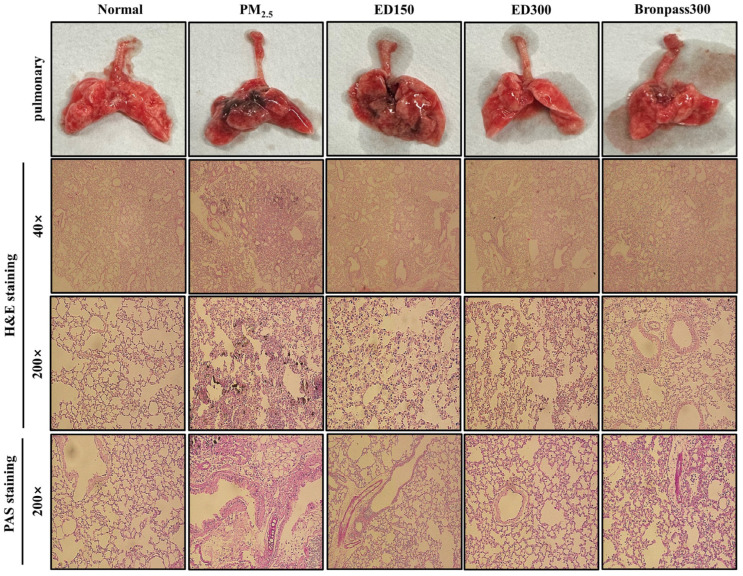
The effect of ED on PM_2.5_-induced mouse pulmonary histopathological changes. The pulmonary sections were stained with H&E or PAS and examined under microscopy (magnification, ×40 and ×200). H&E, hematoxylin and eosin; PAS, periodic acid-Schiff.

To explore the protective effects of ED against PM_2.5_-induced pulmonary damage, we detected histopathological changes in lung tissues through H&E staining. As seen in Figure 5, the PM_2.5_ group exhibited PM_2.5_ deposition, inflammatory cell infiltration, and congestion in the pulmonary cell walls. In contrast, the ED150, ED300, and Bronpass300 groups showed reduced inflammatory cell infiltration. PAS staining confirmed the suppression of mucus secretion, with excess mucus observed in the PM_2.5_ group, whereas this was inhibited in the ED and Bronpass groups (Figure 5).

### 3.5. Effects of ED on Inflammatory Cytokine Levels in the Serum of a PM_2.5_-Induced Pulmonary Inflammation Mouse Model

**Figure 6:**
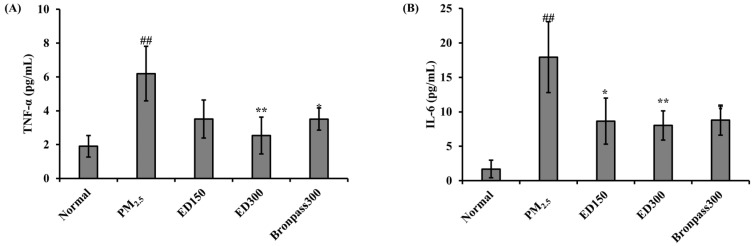
The levels of (**A**) TNF-α and (**B**) IL-6 in serum of PM_2.5_-induced pulmonary inflammation mouse model. All results are shown as the mean ± SD (*n* = 5 per group). ## *p* < 0.01, compared to the normal group. * *p* < 0.05 and ** *p* < 0.01, compared to the PM_2.5_ group.

TNF-α and IL-6 are important mediators of inflammatory responses and play a central role in the pathophysiology of inflammatory diseases. Our results indicated an increase in serum levels of TNF-α and IL-6 in the acute pulmonary model induced by PM_2.5_ (## *p* < 0.01). The ED150, ED300, and Braonpass300 groups decreased the levels of TNF-α and IL-6, with the ED300 group showing the significantly (** *p* < 0.01) highest reduction effect, particularly in the PM_2.5_-induced pulmonary model (Figure 6).

### 3.6. Effects of ED on Inflammatory Cytokine Levels in Lung Tissue of the PM_2.5_-Induced Pulmonary Inflammation Mouse Model

**Figure 7:**
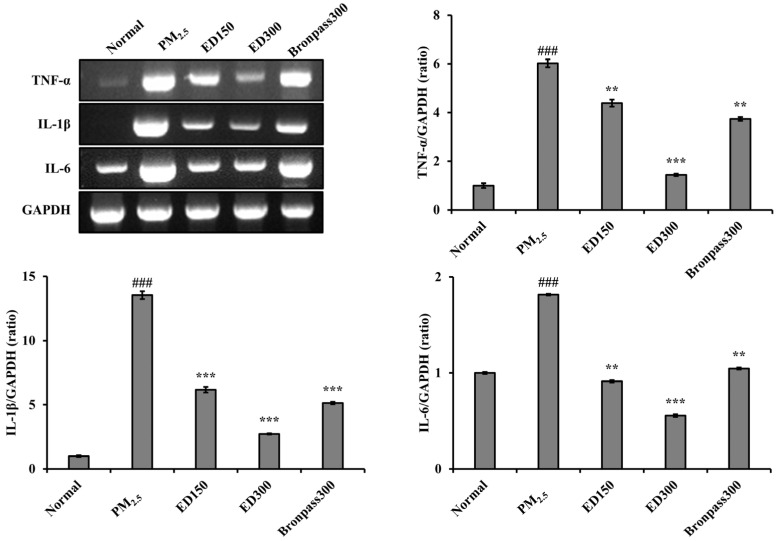
Effect of ED on the mRNA expression levels of *TNF-α*, *IL-1β*, and *IL-6* in pulmonary of PM_2.5_-induced pulmonary inflammation mouse model. RT-PCR analyses analysis using *GAPDH* as the loading control was performed for measurement of *TNF-α*, *IL-1β*, and *IL-6* mRNA expression in pulmonary tissues. The relative mRNA levels of *TNF-α*, *IL-1β*, and *IL-6* were quantified using the Image J program. All results are shown as the mean ± SD (*n* = 5 per group). ### *p* < 0.001, compared to the normal group. ** *p* < 0.01 and *** *p* < 0.001, compared to the PM_2.5_ group.

The observed changes in inflammatory markers in lung tissue of the PM_2.5_-induced pulmonary inflammation mouse model are shown in Figure 7. Exposure to PM_2.5_ was shown to increase the expression levels of inflammatory cytokines (*TNF-α*, *IL-1β*, and *IL-6*) in the lung tissue of mice. Conversely, the ED group effectively decreased the expression levels of inflammatory cytokines, indicating an amelioration of inflammatory responses in the lung tissues.

## 4. Discussion

PM is known to induce various respiratory diseases. Given the current lack of definitive solutions, preventive and therapeutic approaches are essential. Particularly, PM is categorized into PM_10_ and PM_2.5_ based on particle size. As the particle size of PM decreases, it exacerbates lower respiratory tract conditions and increases inflammatory cytokines through the generation of reactive oxygen species (ROS) [33].

The respiratory system is a crucial organ that interfaces with the external environment and possesses a defense mechanism to ward off harmful substances from the surroundings [34]. It comprises mucous membranes that envelop the respiratory tract, and an immune system developed along the respiratory epithelium. The mucus, coating the mucous membranes, is a major component of sputum, consisting of glycoproteins, water, electrolytes, and lipids. Through collaborative action with ciliated cells, it prevents direct contact with the external environment and aids in the removal of foreign bodies or pathogens to the outside. The primary component of mucus, mucin, is produced by goblet cells and mucous cells in submucosal glands. It possesses high viscosity and is primarily composed of heavily glycosylated proteins, acting as potent receptors for carbohydrates. This function allows mucus components to easily bind bacteria composed of carbohydrates in the mucus layer, facilitating their expulsion [35]. However, excessive secretion of mucus can lead to various respiratory conditions. When specific stimuli enter the airways, important glycoproteins such as mucin, a critical component of mucus, are produced [35]. In this context, this study aimed to investigate the anti-inflammatory effects of *E. cava* and *C. indicum* complex extract (ED), which demonstrated anti-inflammatory effects in an OVA-induced asthma model, on *MUC5AC* inhibition in PMA-induced pulmonary epithelial cells and their impact on pulmonary inflammatory responses in a PM_2.5_-induced pulmonary inflammation mouse model.

In the past, most medications for managing inflammatory airway diseases focused on weakening excessive mucus production and secretion in the airways that occur during the onset of these diseases. However, the regulation of mucus secretion and production has become a significant approach to control excessive airway mucus [36]. Therefore, to elucidate the effects of ED and its active component dieckol on the expression of *MUC5AC*, a gene responsible for airway mucus production, we investigated their effects on PMA-induced *MUC5AC* expression in A549 human pulmonary epithelial cells. Mucin present in inflammatory airway diseases is induced by inflammatory mediators such as PMA. PMA is an inflammatory stimulant that regulates gene transcription, cell growth, and differentiation [37]. Moreover, it can induce the gene expression of inflammatory cytokines, including TNF-α [38]. Specifically, PMA is involved in mucin secretion and is used as a protein kinase C (PKC) activator, inducing the expression of mucin genes, including *MUC5AC*, in human airway cells. *MUC5AC* is a major component of gel-forming mucins in the respiratory system and is induced by the activation of the MAPK cascade under PMA stimulation [39]. In this study, it was found that PMA treatment induced *MUC5AC* mRNA expression, and treatment with ED and dieckol suppressed the upregulated mucin gene expression of *MUC5AC* (Figure 2). Furthermore, the MAPK pathway, including JNK, ERK, and p38, activated by PMA, was dose-dependently inhibited by ED and dieckol treatments (Figure 3). Therefore, excessive mucus secretion induced by PMA stimulation was reduced by ED and dieckol, demonstrating their potential as regulators to maintain appropriate levels of *MUC5AC* as a defense mechanism against external stimuli.

Due to the potential for PM-induced damage to various organs, including the respiratory and cardiovascular systems, and the associated increase in mortality rates [40], the need for the development of treatment methods to treat or prevent respiratory damage caused by PM is becoming more critical. In response to this demand, a mouse model of pulmonary damage induced by PM_2.5_, representing a condition similar to respiratory diseases induced by PM in humans, has been established [1,41]. Development of respiratory protective drugs using natural substances is gaining popularity in PM_2.5_-induced respiratory disease models [1,29,30] due to their relatively low side effects and excellent anti-inflammatory and antioxidant activities derived from natural products [18]. ED, a compound composed of *E. cava* and *C. indicum* extracts, has demonstrated excellent antioxidant and anti-inflammatory activities in previous studies. It was confirmed to effectively inhibit inflammation in an OVA-induced asthma mouse model [29]. Therefore, in this study, we evaluated the pulmonary anti-inflammatory effects of ED using the PM_2.5_-induced pulmonary inflammatory mouse model [1,41]. Particularly, we conducted a comparative analysis using Bronpass tablet, a respiratory disease therapeutic drug composed of natural substances, as the positive control group.

The fundamental mechanisms through which PM exerts biological effects are complex. PM exposure can impact various cell types at different levels of immune regulation [42]. Inflammation is considered a key mechanism in the onset of various PM-induced pulmonary diseases [43]. According to several studies, PM’s endotoxins have been positively associated with the production of *TNF-α, IL-1β*, and *IL-6* [44]. Similarly, under our experimental conditions, PM_2.5_ increased inflammatory responses in the mouse pulmonary, a well-known indicator of pulmonary inflammation, as observed in BALF (Figure 4), and induced histological changes in pulmonary tissues (Figure 5). In contrast, ED treatment was found to inhibit inflammatory cell infiltration and excessive mucus production in BALF and pulmonary tissues. Furthermore, ED significantly decreased the secretion and mRNA expression of *TNF-α, IL-1β*, and *IL-6* in serum and pulmonary tissues (Figure 6 and Figure 7).

These results clearly demonstrate that oral administration of ED at doses of 150 and 300 mg/kg effectively inhibits PM_2.5_-induced inflammatory lung damage in a dose-dependent manner through the inhibition of excessive mucus production and anti-inflammatory activity. Therefore, administering an appropriate dose of ED orally holds high potential for future utilization as a natural medicine or health functional food ingredient for effective improvement of respiratory function. However, this study has several limitations that need to be addressed in future research. While the in vitro and in vivo experimental analyses in this study provide valuable insights, they may not fully replicate the complexity of human respiratory diseases. Hence, caution should be exercised when extrapolating the results to clinical scenarios. Nevertheless, this study primarily focuses on the potential impact of ED extract in inhibiting *MUC5AC* and addressing respiratory issues caused by PM_2.5_. Further research is needed to investigate the broad-ranging effects of ED extract on other respiratory parameters, such as strengthening the pulmonary barrier, through additional studies.

## 5. Conclusions

**Figure 8:**
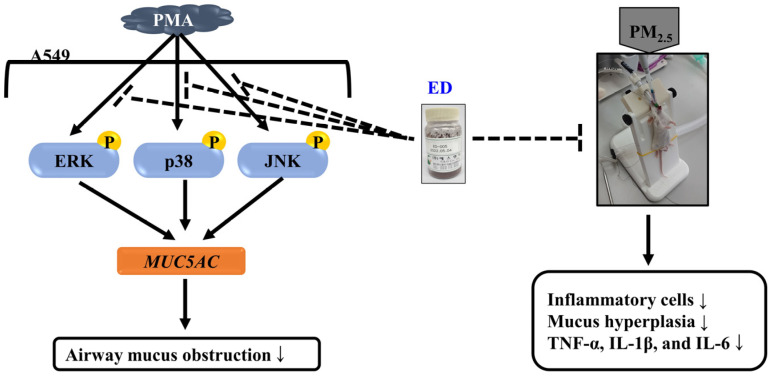
Proposed mechanism of ED in PMA-treated lung epithelial cells and PM_2.5_-induced pulmonary inflammation in mice. ↓: decrease or inhibition.

In this study, we confirmed that ED inhibits excessive mucus production and improves PM_2.5_-induced pulmonary inflammation through the suppression of *MUC5AC* expression. ED exhibited non-cytotoxic effects and reduced *MUC5AC* mRNA expression in PMA-stimulated A549 cells. The downregulation of phosphorylation of JNK, ERK, and p38 in PMA-stimulated A549 cells by ED implies its role as a natural compound that inhibits *MUC5AC* expression. Furthermore, in a PM_2.5_-induced pulmonary inflammation mouse model, ED reduced inflammatory cells in BALF, and histological analysis indicated decreased infiltration of inflammatory cells and mucus formation in mouse pulmonary tissue. Additionally, ED lowered the serum levels and mRNA expression of inflammatory cytokines such as *TNF-α, IL-1β*, and *IL-6* in both serum and pulmonary tissue (Figure 8). These findings suggest that administering an appropriate dose of ED orally holds high potential as a new natural medicine or functional food ingredient for effectively improving respiratory function in the future.

## Figures and Tables

**Table 1:** The sequences of the primers used in this study.

Gene	Size (bp)	Sequence	Origin
*MUC5AC*	458	F ^1^: 5′-TGATCATCCAGCAGGGCT-3′ R ^2^: 5′-CCGAGCTCAGAGGACATATGGG-3′	Human
*Rig/S15*	361	F: 5′-TTCCGCAAGTTCACCTACC-3′ R: 5′-CGGGCCGGCCATGCTTTACG-3′	Human
*TNF-α*	390	F: 5′-TTCGAGTGACAAGCCTGTAGC-3′ R: 5′-AGATTGACCTCAGCGCTGAGT-3′	Mouse
*IL-1β*	385	F: 5′-CATATGAGCTGAAAGCTCTCCA-3′ R: 5′-GACACAGATTCCATGGTGAAGTC-3′	Mouse
*IL-6*	435	F: 5′-GGAGGCTTAAITACACATGTT-3′ R: 5′-TGATTCAAGATGAATTGGAT-3′	Mouse
*GAPDH*	378	F: 5′-CCAGTATGACTCCACTCACG-3′ R: 5′-CCTTCCACAATGCCAAGTT-3′	Mouse

^1^ Forward; ^2^ reverse.

## Data Availability

Data are contained within the article and Supplementary Materials.

## Supplementary Materials

The following supporting information can be downloaded at: https://www.mdpi.com/article/10.3390/pharmaceutics15112621/s1, Figure S1: Original photographs for the blots of each protein marker of Figure 3.

## Abbreviations

PM: particulate matter; OECD: organization for economic cooperation and development; CO: carbon monoxide; DEPs: diesel exhaust particles; COPD: chronic obstructive pulmonary disease; MUC: mucin; BALF: bronchoalveolar lavage fluid; NIST: national institute of standards and technology; PBS: phosphate-buffered saline; FBS: fetal bovine serum; PMA: phorbol 12-myristate 13-acetate; MTT: 3-(4,5-Dimethylthiazol-2-yl)-2,5-diphenyltetrazolium bromide; RT-PCR: reverse transcription-polymerase chain reaction; DW: distilled water; H&E: hematoxylin and eosin; PAS: periodic acid-Schiff; MAPKs: mitogen activated protein kinases; ROS: reactive oxygen species; ED: *E. cava* and *C. indicum* complex extract; PKC: protein kinase C; IL: interleukin; TNF-α: tumor necrosis factor-α.
