# Supplementary material for: Effect of E. cava and C. indicum Complex Extract on Phorbol 12-Myristate 13-Acetate (PMA)-Stimulated Inflammatory Response in Human Pulmonary Epithelial Cells and Particulate Matter (PM)2.5-Induced Pulmonary Inflammation in Mice
Source: Pharmaceutics. 2023 Nov 13;15(11):2621. doi: 10.3390/pharmaceutics15112621 (PMC10674792; doi:10.3390/pharmaceutics15112621)

Original Images for Blots/Gels

Supplementary Figure S1. Original photographs for the blots of each protein marker of Figure 3(A-B).

Figure 3

(A)

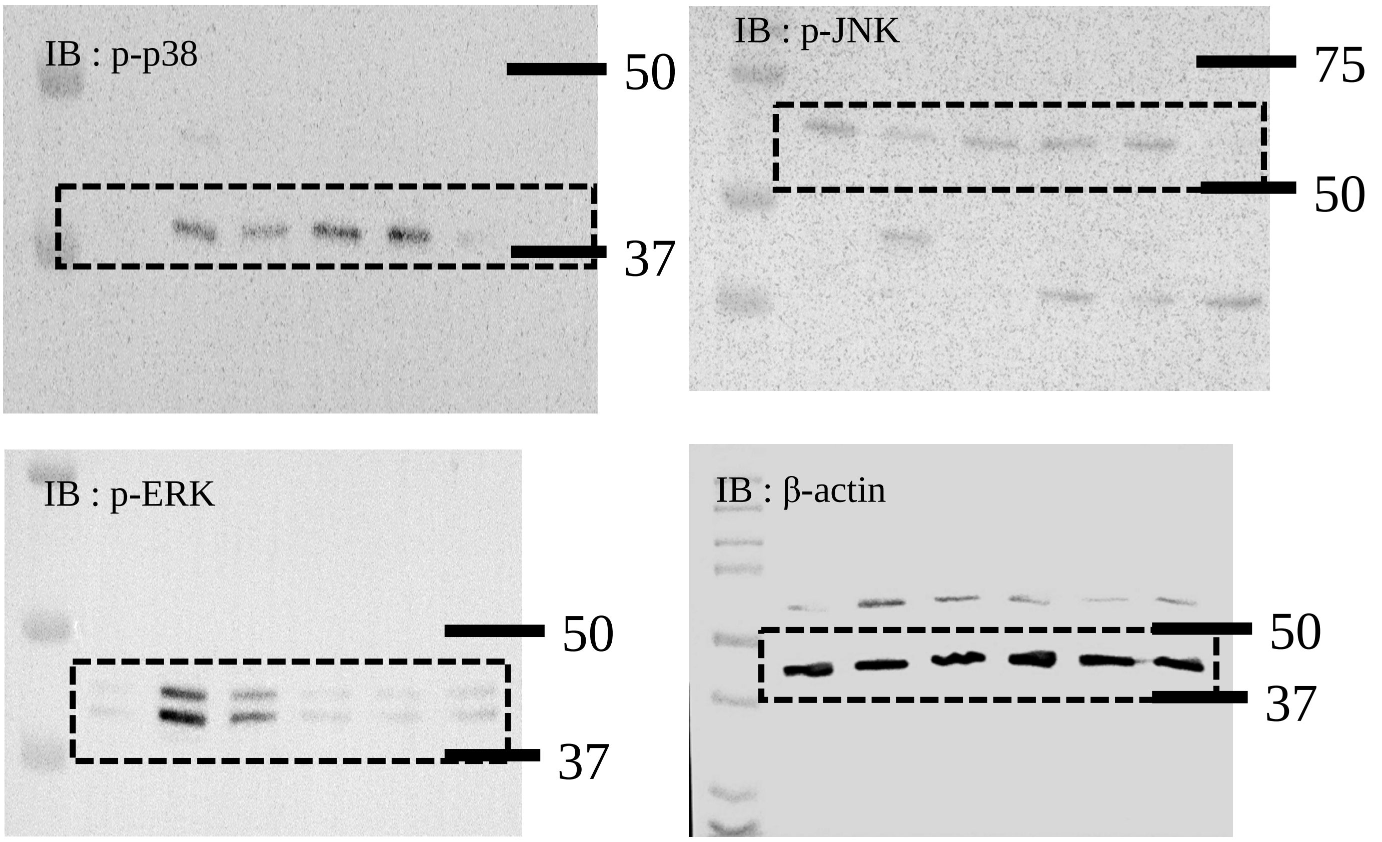

(B)

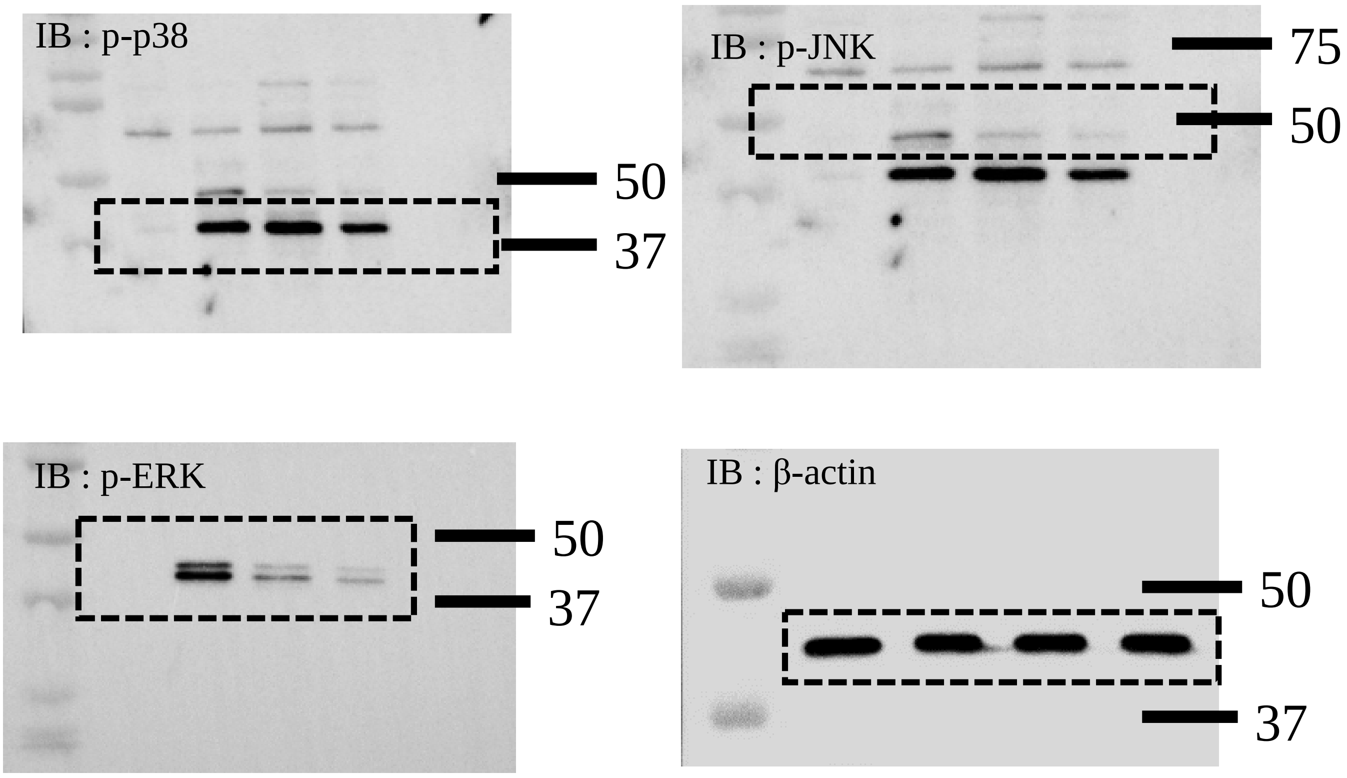

Supplement: Supplementary file 1 [file pharmaceutics-15-02621-s001.zip › pharmaceutics-2680121-supplementary.pdf]
